# Tuning the Intrinsic Properties of Carbon Nitride for High Quantum Yield Photocatalytic Hydrogen Production

**DOI:** 10.1002/advs.201800820

**Published:** 2018-08-14

**Authors:** Mohammad Z. Rahman, Kenneth Davey, C. Buddie Mullins

**Affiliations:** ^1^ John J. Mcketta Department of Chemical Engineering & Department of Chemistry Center for Electrochemistry Texas Materials Institute University of Texas at Austin Austin TX 78712‐1589 USA; ^2^ School of Chemical Engineering The University of Adelaide Adelaide SA 5005 Australia

**Keywords:** carbon nitride, molecular engineering, photocatalysts, solar fuel

## Abstract

The low quantum yield of photocatalytic hydrogen production in carbon nitride (CN) has been improved upon via the modulation of both the extrinsic and intrinsic properties of the material. Although the modification of extrinsic properties has been widely investigated in the past, recently there has been growing interest in the alteration of intrinsic properties. Refining the intrinsic properties of CN provides flexibility in controlling the charge transport and selectivity in photoredox reactions, and therefore makes available a pathway toward superior photocatalytic performance. An analysis of recent progress in tuning the intrinsic photophysical properties of CN facilitates an assessment of the goals, achievements, and gaps. This article is intended to serve this purpose. Therefore, selected techniques and mechanisms of the tuning of intrinsic properties of CN are critically discussed here. This article concludes with a recommendation of the issues that need to be considered for the further enhancement in the quantum efficiency of CN photocatalysts.

## 1 Introduction

Photocatalytic hydrogen production from water is a promising but challenging alternative to augment the energy problem in the post fossil fuel era.^1^ In this process, a photocatalyst is used to produce hydrogen through the interaction of sunlight and water.^2^, ^3^ The photocatalyst transforms absorbed solar photons into excitons (i.e., excited electrons and holes) to induce essential redox reactions for releasing hydrogen from water.^4^, ^5^

Since the first report of photoelectrocatalytic water splitting on TiO_2_ in 1972,^6^ a mix of metal and metal‐free photocatalysts has been cataloged.^5^, ^7^, ^8^ Despite noticeable success, designing of a cost‐effective stand‐alone photocatalytic reactor with a practically compatible technology does not seem to be a near term reality without the discovery of a truly scalable photocatalyst. Stable, low‐cost, and earth‐abundant raw materials are a primary concern, and carbon nitrides (CNs) have the potential to fill this gap.^9^, ^10^

The interest with CN began due to its remarkable physicochemical properties for photocatalytic solar fuel production via water splitting. These salient features include: appropriate energy levels (band gap ≈2.7 eV; lowest unoccupied molecular orbital (LUMO) ≈ −1.1 V vs standard hydrogen electrode (SHE); highest occupied molecular orbital (HOMO) ≈ +1.6 V vs SHE^9^) straddling the redox potential required (i.e., 0.0 V vs SHE for proton reduction and +1.23 V vs SHE for water oxidation^2^) for water splitting, visible‐light photons absorption, strong stability in solutions with pH = 0–14, and easy synthesis from readily available inexpensive precursors.

However, the low quantum yield of hydrogen in carbon nitride is a great shortcoming with regard to becoming an industrially scalable photocatalyst. The narrow absorption edge in visible light, high charge transfer resistance, high rate of charge carrier recombination, inefficient utilization of charge carriers, low catalytic active surface area, etc., are the primary reasons for this.^11^, ^12^ These problems are dealt with by modifying the electronic structure, nanostructure, crystal structure, and constructing heterostructures.^13^, ^14^

The conventional approaches to enhancing the photocatalytic activities of CN include texturization for increased surface area, copolymerization with organic/inorganic dopants for tuned electro‐optical properties, hybridization with other materials for enhanced charge separation, and homo‐heterojunction concepts for improving interfacial charge transfer.^15^ These approaches of geometrical structuring and/or interfacing with other materials have proven useful for enhancing the extrinsic activities of CN.^16^ However, the manipulation of extrinsic activities has yielded limited improvements in the apparent quantum efficiency (AQE ≤ 10%).^17^ This shortcoming has therefore led to efforts regarding improvements in the intrinsic activity of CN. By intrinsic properties we mean the molecular tunability of chemical composition and structure of the catalyst materials.

**Scheme 1:**
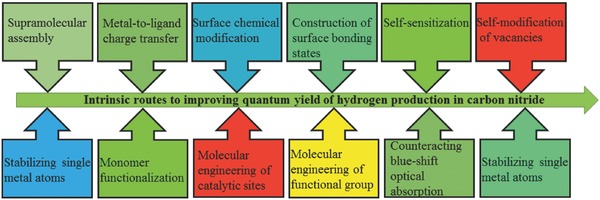
Intrinsic routes to improving quantum yield of hydrogen production in carbon nitride.

The molecular tuning of intrinsic activities has been facilitated by the advancement in computational quantum chemistry to optimize the structure, compositions, and electrochemical surface states at the molecular level.^18^ Additionally, the in‐depth mechanistic understanding of the nature of photochemical, electrochemical, and electro‐optical processes has been improved through advanced surface electrochemistry, material science, and state‐of‐the‐art nanotechnology. This combined (theory and experiment) approach therefore assists in the design and modification of CN with advantageous properties to approach maximum activities. In recent years, molecular tuning of the intrinsic catalytic activities of CN photocatalysts was attained through quite a number of different approaches. For example, supramolecular assembly, metal‐to‐ligand charge transfer, surface chemical modification, construction of surface bonding states, self‐sensitization and homogeneous self‐modification of vacancies, stabilizing single metal atoms, monomer functionalization, identification and molecular engineering of functional groups as potential catalytic sites, increasing crystallinity, counteracting blueshift optical absorption, etc., are worth mentioning (see **Scheme** **1**). With intrinsic molecular tunability, the AQE of standalone CN photocatalysts reached as high as 60% under simulated solar irradiation.^19^

Several excellent reviews^8^, ^15^ have reported the approaches for enhancing extrinsic activity along with the latest progress and the history, synthesis, properties, and applications of CN. However, a critical analysis of the research involved in tuning of the intrinsic activities of CN as a photocatalyst, to the best of our knowledge, has yet to be reported. Therefore, in this review, our focus is to critically present and summarize selected research findings regarding the modification of intrinsic photophysical properties of CN.

This article is organized as follows: after a concise introduction, a briefing on the state‐of‐the‐art CN is highlighted; we then critically judge and discuss research that has an impact regarding the tuning intrinsic properties on the photocatalytic activity of CN. We conclude with identification of the intellectual and research gaps along with recommendations for future research directions regarding CN photocatalysis.

## 2 A Brief Overview of Carbon Nitrides

In Justus von Liebig's era, graphitic carbon nitride (GCN) was known as melon, a 1D polymer of heptazine that is bridged by secondary amines, connected with neighboring polymer strands via hydrogen bonds, and stacked in a 2D layered fashion through π–π interactions.^20^ GCN is also reported as a 2D layered structure with tertiary amines bridging all the heptazine units^21^ or a poly(heptazine imide) hexagonal network^22^ where each heptazine forms three secondary amine bonds with neighboring heptazine units. The term “GCN” was postulated by several authors due to the structural resemblance to graphite.^21^, ^23^ There are two models, based on triazine (C_3_N_3_) and heptazine (C_6_N_7_) moieties, which have emerged over the years to account for the geometry and stoichiometry of GCN.^22^, ^24^ The size of the nitrogen‐linked aromatic moieties (sp^2^‐hybridized carbon and nitrogen atoms) is the distinguisher between these two models. The density functional theory (DFT) calculations revealed that the valence bands (VBs) are composed of nitrogen P_z_ orbitals while the conduction bands (CBs) are composed of carbon P_z_ orbitals.^9^ Therefore nitrogen supplies holes to act as oxidation reaction sites, and carbon supplies electrons to act as reduction reaction sites.

**Figure 1:**
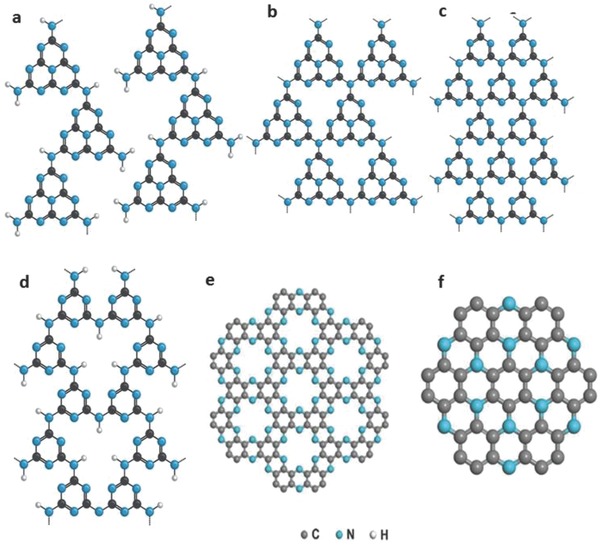
Molecular structure of a) melon, b) heptazine‐based PCN, c) triazine‐based PCN, d) polytriazineimide (PTI), e) C_2_N, and f) C_3_N. a–d) Reproduced with permission.^22^ Copyright 2016, Elsevier. e) Reproduced with permission.^28^ Copyright 2015, Nature Publishing Group. f) Reproduced with permission.^29^ Copyright 2016, PNAS.

Besides polycrystalline GCN, amorphous carbon nitride (ACN) has also been reported to be a promising photocatalyst.^25^, ^26^, ^27^ Varying the C/N ratio, two eminent derivatives of carbon nitride, namely, C_2_N^28^ and C_3_N^29^ have been reported, which might also possibly be used as a photocatalyst. The C_2_N contains many irregular holes in its crystalline structure while C_3_N consists of a hole‐free 2D honeycomb lattice. The C_2_N has a direct band gap of 1.96 eV and the C_3_N has an indirect intrinsic band gap of 0.39 eV. The band gap of C_3_N is tunable (2.74–1.57 eV) with a morphological shift from nanosheets to quantum dots.^30^ However, the practical demonstration of C_2_N and C_3_N as a photocatalyst is still in a nascent stage. Therefore, we will limit our discussion in this contribution mainly to GCN and ACN. The molecular evolution of melon to different versions of CN and its derivatives is depicted in **Figure** **1**.

## 3 Suitability of Carbon Nitrides as a Photocatalyst

Photocatalytic hydrogen production via water splitting is comprised of two half reactions: the water reduction reaction for evolving hydrogen and the water oxidation reaction to evolve oxygen. Therefore, a semiconductor photocatalyst should meet the thermodynamic requirements for the standard redox potentials (see Equations (1)–(3)) for water splitting.

(1) $$\begin{array}{c} 2H_{2}O\to 2H_{2}+O_{2}\;\;\;2.46\;\;\mathrm{eV} \end{array}$$

(2) $$\begin{array}{c} 2H_{2}O\to O_{2}+4H^{+}+4e^{-}\;\;\;0.81\;V \end{array}$$

(3) $$\begin{array}{c} 2H_{2}O+2e^{-}\to 2H_{2}+2{\mathrm{OH}}^{-}\;\;\;-0.42\;\;\;V \end{array}$$

Therefore, the semiconductor photocatalyst should have a minimum band gap of 1.23 eV with the potentials for reactive electrons and holes to be equal to or greater than −0.42 V and equal to or less than 0.81 V at pH = 7, respectively. This applies only for the standard condition of zero overpotential and zero reorganization energies for interfacial charge transfer reactions, and therefore is not practical for hydrogen production via water splitting. The sum of the energy both for oxidation and reduction reactions is 0.4 V (see Equations (2) and (3)). Therefore, the band gap has to be at least in the range of ≈2.0 eV.

Carbon nitride fulfills these requirements. It has suitable energy levels (band gap ≈2.7 eV; LUMO ≈ −1.1 V vs SHE; HOMO ≈ +1.6 V vs SHE^9^) straddling the redox potential required (i.e., 0.0 V vs SHE for proton reduction and +1.23 V vs SHE for water oxidation^2^) for water splitting.^8^, ^15^, ^31^ These HOMO and LUMO values are predictions from computational results using DFT. The experimental values for the band gap, and corresponding CB and VB positions, vary between ≈2–3 eV, −0.5 to −1.3 V versus SHE, and 1.3–1.8 V versus SHE, respectively.^11^, ^32^, ^33^ The experimental CB position is more negative than the water reduction potential, and the VB position is more positive. These properties are favorable therefore for the production of hydrogen through a water splitting half reaction in the presence of sacrificial agents (e.g., triethanolamine (TEOA), methanol, and lactic acid) or overall water splitting with the aid of a suitable co‐catalyst (e.g., Pt, CoO*_x_*, CoP, and PtO*_x_*).^34^, ^35^

In 2009, Wang et al.^9^ reported melon as a metal‐free photocatalyst together with an sacrificial electron donor (SED) for hydrogen production for the first time. Soon after their results drew significant attention from researchers and became the subject of a vibrant field of research in photocatalytic water splitting. Consequently, in recent years many derivatives of melon have been reported as photocatalysts.

From Equations (1)–(3), it can be understood that hydrogen can be produced either via overall water splitting (production of hydrogen and oxygen at the same time) or a water‐reduction half‐reaction while suppressing the water‐oxidation half‐reaction. This can be accomplished using an appropriate SED. Therefore, most research has been directed toward the water‐reduction half‐reaction in the presence of an SED. Although it is highly desirable, a standalone carbon nitride has not been reported for overall water splitting in the absence of an SED. However, there are few reports with sacrificial electron donors and acceptors for overall water splitting.^34^

## 4 Selective Techniques for Tuning Intrinsic Properties of CN

Advances in synthetic chemistry and computational quantum chemistry have led to the preferential molecular tuning of intrinsic catalytic activities of CN that can be used to overcome low quantum yield and enhance photocatalytic hydrogen production via water splitting. A critical review of selected research that has been directed to achieve high apparent quantum efficiency of polymeric carbon nitride (PCN) is discussed below.

### 4.1 Metal Coordination by Preferential Metal Complexion

The band gap of PCN (≈2.75 eV) is sufficient to over‐energize most of the redox reactions. Water splitting ideally requires 1.23 eV energy. Research has therefore been directed toward narrowing the band gap and increasing photon absorption into the visible range of the electromagnetic spectrum. This has been largely done by doping heteroatoms. However, doping adversely effects the redox reactions by influencing charge carrier recombination.

Creating metal complexes was found as a possible way to address this problem with doping. Modulation of electro‐optical properties of porphyrins, chlorophyll, hemin, etc., after metal complexation with Mg, Fe, Ni, Cu, and Zn ions is a “good” example. Metal co‐ordination significantly alters the spectrochemical properties and provides additional absorption bands. PCN has high nitrogen content. The six lone‐pair electrons from the nitrogen atoms provide ideal sites for inclusion of metal cations or atoms. Therefore, the formation of metal complexes (e.g., Fe^3+^, Co^2+^, Co^3+^, and Pt^2+^) as an alternative to doping has also been suggested for PCN.^36^

**Figure 2:**
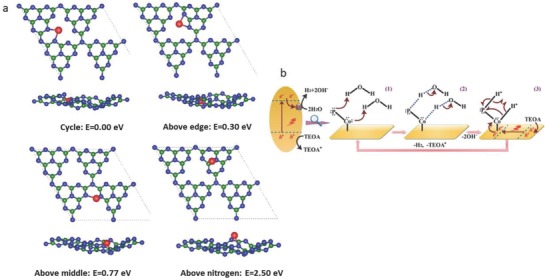
a) Top and side views of Co coordinated in different sites of the GCN matrix after geometry optimization. Green, blue, and red represent: C, N, and Co atoms, respectively. Bottom values indicate the relative binding energies of different atomic configurations. Reproduced with permission.^37^ Copyright 2017, American Chemical Society. b) A dual molecular synergistic reaction mechanism of photocatalytic hydrogen production by CoP/GCN. Upon absorption of visible light, electron accumulated in conduction band (CB) transfers to CoP and then reduces H_2_O to release H_2_, and the holes accumulated in valence band are consumed by triethanolamine (TEOA). In step 1, P and Co atoms adsorb one proton each from a H_2_O molecule by their lone pair electrons to create a transition state of two H_2_O molecules adsorption. In step 2, a dual protonation state is created by transferring two electrons from H—O bond and releases two OH**^−^**. TEOA injects electrons into CB of GCN to consume two holes and two electrons transfer to CB of Co atom. In step 3, P–H and Co–H covalent bonds induce hemolytic reactions for coupling two protons to form a H_2_ molecule. Reproduced with permission.^38^ Copyright 2017, Wiley‐VCH.

Zheng et al.^37^ recently reported insights into coordination ability, catalytic nature, and activity origin for different 3d transition metals (Cr, Mn, Fe, Co, Ni, Cu, and Zn) with an emphasis on Co—C—N coordination in GCN. The results showed that the Co connected with the two adjacent pyridinic‐N atoms from two separate triazine units was stable, exhibited a high rate of charge transfer (≈0.84 e‐ based on Bader charge analysis), and gave good catalytic activity. These results from geometry optimization with distinctively different positions of Co in GCN are shown in **Figure** **2**a.

In contrast to creating single metal complexes, creating metal centers using compounds within the PCN framework has also been shown effective in boosting hydrogen production. This is because they provide dual molecular synergistic reaction mechanisms. In a recent study, by creating P—Co—N surface bonding states a significant increase in hydrogen quantum yield (12.4%) was found at 420 nm.^38^ In this P—Co—N system, Co—N bonds were reported to improve the charge transfer and separation between the host catalyst (polymeric carbon nitride) and co‐catalyst (CoP); while, P—Co bonds formed a Co‐hydride and protonated P‐pendant moieties with high electron density to make adjacent Co and P atoms act as dual proton adsorption sites. This Co‐hydride and protonated P‐pendant moieties therefore produced a dual molecular synergistic effect. The photocatalytic reaction mechanism of P—Co—N system is depicted in Figure 2b (see the caption for details).

The activity of the host catalyst upon inclusion of metal cations depends on the speciation (single atoms, dimers, clusters, or nanoparticles), the nature of the distribution, and the oxidation states of the metal atoms within the host. “Best” performance was achieved while the metal atoms were distributed in single‐atom fashion—rather than in the formation of cluster/nanoparticles.^39^ However, realization of stabilization of single atoms on a given carrier (host catalyst) poses a challenge to synthesis. To avoid agglomeration, two criteria need to be maintained concomitantly: 1) increase cohesive energy of the metal species with the carrier, and 2) do so without diminishing the activity via changes in the electronic configuration. Maintaining these requires the carrier to be sufficiently flexible to have sufficient interactions with the isolated metal centers. In a practical demonstration of hosting a single atom of different metals, recently Chen et al. compared five metals (Pd, Ag, Ir, Pt, and Au) in PCN.^39^ Their findings suggested that metal atoms increasingly form nanoparticles in the case of bulk PCN, due to low surface area, while maintaining a single atom/dimer nature in nanosheets as well as in mesoporous structures.

**Figure 3:**
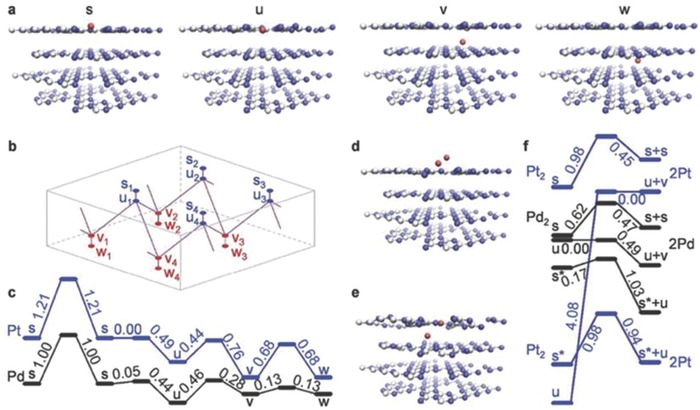
a) DFT optimized incorporation of metal atoms in g‐C_3_N_4_ (M = red, C = white, N = blue), showing the four identified equilibrium positions (s, u, v, w) of metal atoms. b) Identified transition paths of metal atoms inside the surface and subsurface layers of g‐C_3_N_4_. c) Corresponding energy profiles for translocation of Pt and Pd atoms along these paths. d,e) Identified equilibrium positions of metal dimers d) above and e) below the surface layer of g‐C_3_N_4_. f) Energy profiles for the association of metal dimers and their decomposition into pairs of atoms located in neighboring s + s, s* + u, or u + v positions (s* denotes s‐sites occupied by hydrogenated, therefore, immobile metal atoms or dimers). Reproduced with permission.^39^ Copyright 2017, Wiley‐VCH.

Mesoporous structures were shown to be most favorable for single‐atom stabilization implying the involvement of defects in anchoring the single atoms. When it is a matter of loading (wt%) of metal atoms, copolymerization appears to be most efficient (≈100 wt%), and wet deposition showed the greatest tendency for nanoparticle formation. DFT calculations for Pt and Pd (representing two extreme cases) identified four metastable positions of the isolated metal atoms, which are denoted by s, u, v, and w in **Figure** **3**, above and below the interstices between tri‐s‐triazine units in the top layers of PCN. Based on computed energy, Pt and Pd atoms in u positions are expected to be the most stable.

Design of appropriate comonomers with diverse chemical composition and structure allows the modification of the band structure and optoelectronic properties of carbon nitride to improve the photocatalytic activity and selectivity of carbon nitride. For example, copolymerization of barbituric acid with the carbon nitride precursor through a Schiff base reaction extends the optical absorption of the polymer to cover more of the visible light range, and therefore leads to an enhanced performance with carbon nitride.^40^ Zhang et al. advanced this strategy by employing a variety of new monomer building blocks with the desired compositions and electronic structures for chemical incorporation into the conjugated polymeric network of carbon nitride.^41^ They synthesized organic molecules bearing amino and/or cyano functionalities to integrate them directly into the carbon nitride polymers to alter the physical and chemical properties. The photocatalytic properties of carbon nitride originate from its π‐conjugated system due to the sp^2^ hybridization of C and N. It has therefore opened a new avenue to extend the delocalization of the p electrons and change the intrinsic semiconductor properties by grafting aromatic groups on the surface of carbon nitride. Zhang et al.^41^ have shown that the incorporation of phenylene groups into carbon nitride modifies the p‐electron delocalization in the conjugated system and thus changes the intrinsic optical/electronic properties of the resulting polymers. They have observed a remarkable redshift of the optical absorption from about 460 nm to about 700 nm.

### 4.2 Engineering of Defects with Functional Groups

Lack of purification methods made synthesis of a defect‐free CN difficult. Calcination of the monomer(s) precursor(s) of CN produces intermediates, namely, biuret, cyanuric acid, ammeline, and ammelide, which are responsible for the defects in the end product.^42^ There is a sensitive thermodynamic balance between defect formation and the associated impact in catalyzing functionalities. While it is generally accepted that defects are the recombination/trap centers for photogenerated electron–hole pairs, some recent studies show a positive impact of defects in photocatalytic performance. For example, molecular engineering of cyanamide “defects” and identification of catalytically relevant functional groups in cyanamide derived amorphous melon were demonstrated to be effective for enhancing hydrogen quantum yield.^43^

Incorporation of the cyanamide moiety (—NCN—) or oxygen‐bearing functional groups (—O—, —OH, —COOH) in heptazine molecules has resulted in a novel idea for surface termination and defects to become active catalytic sites. These functional groups are generated in situ due to incomplete heptazine cyclization/thermal depolymerization, and the presence of oxygen impurities in the precursors (i.e., urea and ureidomelamine), or oxygen contamination through trace water during high‐temperature syntheses in air. These functional groups provide ligating linkages to the co‐catalyst centers and built‐in electrostatic potential differences across the heptazine polymer. This enhances efficient transfer and migration of photogenerated charge carriers from the heptazine core to the co‐catalyst for proton reduction to take place.

**Figure 4:**
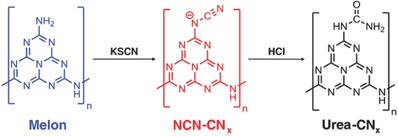
Reaction scheme of inserting oxygen‐functional group in carbon nitride. Reproduced with permission.^44^ Copyright 2017, Wiley‐VCH.

The insertion of oxygen‐bearing functional groups into the heptazine structure (the molecular synthesis of inserting oxygen‐functional group is shown in **Figure** **4**) was reported to act as sites for redox reactions, expedite intermolecular interactions, augment overall reaction kinetics, strengthen catalyst‐substrate affinity, modify carrier dynamics, and enable strong photocatalyst/co‐catalyst interactions. As a result, replacement of amine groups in heptazine‐based polymer melon by oxygen‐bearing groups (i.e., urea) was reported to reach an AQE of 18%.^44^ Compared to nitrogen‐rich precursors (e.g., cyanamide, dicyandiamide, and melamine), CN synthesized from oxygen‐rich urea has demonstrated^45^ a high AQE of 26.5%. It was also shown that C≡N groups can be used to induce two different conductivities within the PCN to build a p–n homojunction.^46^ Recently, Ou and co‐workers reported a selenium and cyanamide‐functionalized heptazine‐based melon (DA‐HM) as a unique bioinspired donor–acceptor light harvester. The DA‐HM accommodates the combination of the photosystem and electron shuttle in a single species, with both n and p‐type conductivities, which endows it with a high efficiency for the transfer and separation of photoexcited charge carriers, resulting in an apparent quantum yield (AQY) of 19.4% at 405 nm.^47^ Engineering of carbon vacancies through steam forming delamination of carbon nitride resulted in an apparent quantum yield of 11.3% at 405 nm.^48^

Research has expanded to mimic the light‐dependent photosynthesis reactions in thylakoid membranes in plants, in which phospholipids play a vital role in the transfer of electrons in electron‐transport chains, and the pumping of protons to drive adenosine triphosphate synthesis, and to act as a mediator in carbon fixation and in converting solar energy into chemical energy in the form of sugars.

These phosphate‐involving natural photosynthesis reactions (known also as phosphorylation) were mimicked to enhance hydrogen production in PCN. It has been shown that an optimal addition of K_2_HPO_4_ in the reaction system can significantly boost the hydrogen production rate (947 µmol h^−1^ with an AQE of 26.1%).^49^ The addition of phosphate facilitates proton transfer in reaction solutions, provides synergy for enhanced proton reduction, and improves hole oxidation that leads to a Calvin‐cycle‐like hydrogen production pathway.

### 4.3 Self‐Modification for Extended Absorption of Visible Light

CN only absorbs in the wavelength region from 420 to 460 nm. Thus, a critical issue is achieving a high quantum yield. Research has been conducted regarding extending the absorption edge of CN that can be categorized as a “third‐party” assisted or self‐assisted method. The third‐party‐assisted approach (i.e., doping heteroatoms) appears in almost all previous reviews on CN. Here self‐assisted approaches will be considered, which include homogenous self‐modification with nitrogen vacancies, homogenous amorphization, supramolecular assembly, self‐sensitization, co‐sensitization, etc.^25^, ^50^, ^51^, ^52^, ^53^

**Figure 5:**
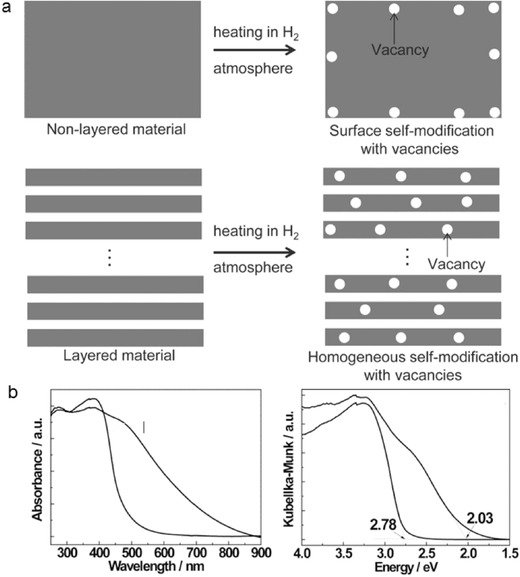
a) Schematic of surface self‐modification and homogeneous self‐modification with vacancies in H_2_ atmosphere. b) UV–vis absorption spectra and Kubelka–Munk plot for band‐gap calculation. Reproduced with permission.^50^ Copyright 2014, Wiley‐VCH.

Homogeneous self‐modification with nitrogen vacancies was realized by heating melon in a hydrogen atmosphere in which hydrogen acted as a reducing agent to reduce terminal amine groups to realize self‐modification (see **Figure** **5**).^50^ Nitrogen vacancies led to an up‐shift of the valence band and a down‐shift of the conduction band, resulting in a band gap narrowing of 0.75 eV (2.78 eV/446 nm to 2.03 eV/610 nm) corresponding to a 164 nm extension of the absorption edge. The hydrogen‐bonded strands of polymeric melon with NH_2_/NH units cohere through weak van der Waals (vdW) forces, while there are strong covalent C–N bonds in each layer. The covalent bonds are more resistant to rupture than vdW and hydrogen bonds that offer a possibility of disrupting long‐range, but keeping short‐range, atomic order for homogeneous amorphization upon heating PCN at elevated temperature. Kang et al. reported ACN with a band gap of 1.90 eV.^25^ Following this, we worked on ameliorating the charge transfer kinetics of ACN to overcome its low quantum yield (<0.5%) for hydrogen production.^54^

CN obtained by supramolecular aggregation followed by ionic melt polycondensation was shown to capture photons beyond 650 nm, while a photochemical synthesis of carbon nitride extends the absorption edge to 735 nm.^51^, ^55^, ^56^ Recently, we have reported carbon nitride nanosheets with redshifted photon absorption edge with high AQE (16%).^33^ Previously, all nanosheets of carbon nitride exhibited a blueshift in the optical absorption, which detrimentally impacted the performance.

The leading edge for light absorption of a semiconductor photocatalyst can be extended by using chromophore dyes. Coupling of dyes can facilitate the absorption of low‐energy photons by a host semiconductor. For example, a microsphere core of oxygen‐containing CN, and self‐sensitized surfaces through covalently linked polymeric triazine dyes, exhibited hydrogen production up to 600 nm with excellent stability for more than 100 h of reaction.^52^ A panchromatic light responsive carbon nitride photocatalyst, sensitized with two different dyes of complementary absorption spectra, yielded hydrogen up to 700 nm monochromatic light irradiation.^53^

**Figure 6:**
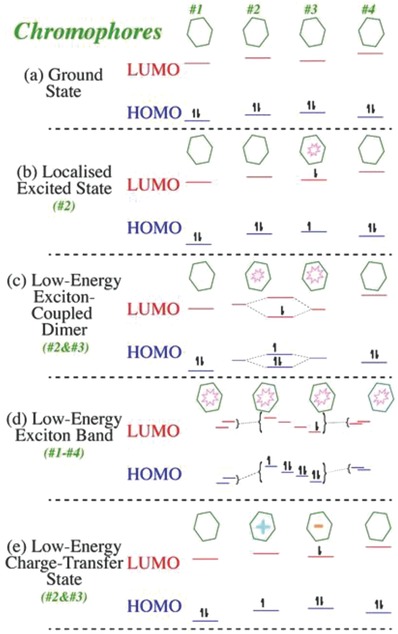
Low‐energy states absorption scenario of chromophores; a) the ground states, and b–e) various possibilities for low‐energy excited states, either localized on one chromophore, associated with exciton coupled dimers or bands, or arising through charge transfer. Reproduced with permission.^57^ Copyright 2016, Elsevier.

**Figure 7:**
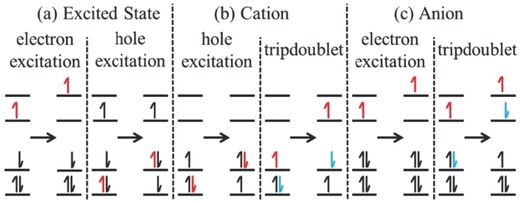
Possible low‐energy hole excitations, electron excitations, and/or tripdoublet excitations of a photoactive species: being a) an excited state, b) a cation, or c) an anion. Electrons directly affected by the transition are highlighted in red or blue; tripdoublet transitions are low‐energy double excitations while the remainder are single excitations. Reproduced with permission.^57^ Copyright 2016, Elsevier.

Low‐energy light absorption from coupling of dyes may arise from one of the four following processes,^57^ as depicted in **Figures** **6** and **7**: i) individual chromophores having low‐energy excited states and interactions with a specific binding site, ii) excitonically coupled assemblies of chromophores to low‐energy excited states, iii) transition of charge transfer states allowing the transfer of an electron from chromophore to chromophore; and iv) excitation of species arising from previous absorption due to creation of an excited state, or excitation of anion and cation, or formation of radical ions with low‐energy tripdoublet bands.

However, which process will dominant depends on many factors: site energies of individual chromophores and excitonic couplings of chromophore pairs; intermolecular and intramolecular geometrics of excited states and its relationship to corresponding ground states; coupling of electronic transitions with phonon modes, interactions between charge transfer states and locally excited states; survivability of an excited state species long enough to be excited by another photon, etc.

Recently, Zhang et al. reported that a co‐condensation of urea and oxamide followed by postcalcination in molten salt could result in crystalline carbon nitride, which improves lateral charge transport and interlayer exciton dissociation. The addition of oxamide decreases the optical band gap from 2.74 to 2.56 eV and enables efficient photochemistry with green light. As a result, they have reported an AQY of 57% at 420 nm and 10% at 525 nm for H_2_ evolution.^58^

### 4.4 Designing Metal‐Free Heterojunctions

Kinetic competition between recombination (surface and/or bulk) and charge transfer reactions determines the number of surface charges that will ultimately take part in interfacial reactions. It is therefore necessary to increase the rate of charge transfer reaction to increase the photoactivity. There are several commonly used techniques to address this issue, including, doping, incorporation of a suitable co‐catalyst with light absorbing semiconductor, and creation of a heterojunction.^59^ Both metal doping (K, Na, Pd, Fe, Cu, W, etc.)^60^ and nonmetal doping (P, S, O, C, N, B, F, etc.)^61^ have been explored to simultaneously tune the crystallographic, textural, and electronic structures of carbon nitride for improving photocatalytic activity. We have not discussed the detailed doping of carbon nitride here as there have already been several excellent reviews on this topic.^62^ Doping could significantly reduce the charge transfer barrier, but it engenders external energy loss due to introduction of carrier recombination centers.^63^ The metal co‐catalyst forms a Schottky‐type heterojunction with the light absorber and generates an internal electric field that is the driving force for charge transfer with low activation energy.^64^ This is the most common way of dealing with charge transfer reactions. However, most active co‐catalyst is a precious metal (e.g., Pt, Au, Ag, Pd, and Rh) and therefore it is not economical for use in hydrogen production via water splitting.

**Figure 8:**
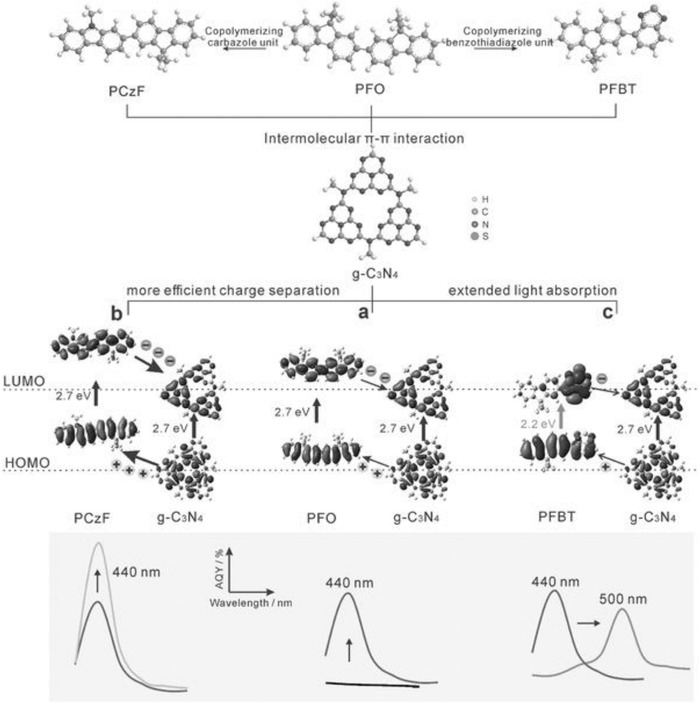
a) Scheme of the molecular design of PHJs between poly‐(9, 9 dioctylfluorene) (PFO) and g‐C_3_N_4_ (GCN) via intermolecular π–π interaction.b) Introduction of electron‐donating carbazole unit for more efficient exciton dissociation in the PHJ. c) The electron‐accepting benzothiadiazole unit was introduced for extended visible light active range. The bottom shows the AQY‐wavelength profiles of the PHJs, demonstrating that the photocatalytic efficiency can be enhanced through molecular design. Reproduced with permission.^70^ Copyright 2017, Wiley‐VCH.

Heterojunctions between two semiconductors facilitate delocalization of charge carriers, retard back recombination of electron–hole pairs,^65^ and thus effectively separate the charge carriers.^66^ Inorganic semiconductor heterojunctions are widely studied while metal‐free heterojunctions are rare because of the limited number of metal‐free semiconductors that can be used as a photocatalyst. Wang and co‐workers demonstrated for the first time an all carbon nitride based isotype heterojunction by a facile band alignment approach.^67^ Other than this, there are only a few examples of metal‐free heterojunctions, such as GCN/C‐dots (AQE of 16%),^68^ CN/h‐BN,^69^ and carbon rings/GCN.^63^ A polymeric heterojunction (PHJ) of GCN with the polyfluorene family was also reported^70^ (see **Figure** **8**). An AQE of 27% for a hydrogen production rate ≈72 µmol h^−1^ was claimed for this PHJ. However, other researchers have reported similar production rates with much lower AQE^12^, ^71^ in almost identical reaction conditions. There is a growing concern therefore regarding reproducibility of the reported record high efficiencies in carbon nitride.^72^ Apart from heterojunctions, a homojunction of GCN by exploiting in situ bond modulation through a defect‐induced self‐functionalization process was reported.^46^ Zhang et al. have recently reported a new benchmark for hydrogen quantum yield. They reported an ionothermal synthesis of triazine‐heptazine‐based co‐frameworks with an AQY of 32% from water and 60% from seawater at 420 nm under visible light irradiation.^19^

**Figure 9:**
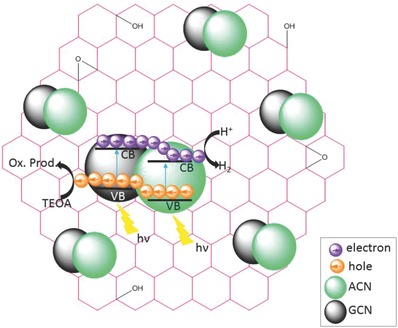
Schematic illustration of charge transfer process for photocatalytic hydrogen in GCN/ACN/rGO. The band offsets between GCN and ACN help in delocalizing the electrons in conduction band of ACN while holes in the valence band of GCN. rGO enhances the transfer of surface electrons to water for proton reduction. Reproduced with permission.^27^ Copyright 2017, the Royal Society of Chemistry.

Recently, for the first time, a ternary metal‐free heterojunction was created (**Figure** **9**) by combining GCN, ACN, and rGO (reduced graphene oxide), which demonstrated a high hydrogen production rate.^27^ In‐plane stitching of 2D domains of GCN, ACN, and rGO with similar aromatic structure induces an intrinsic driving force for delocalization of photocarriers around the photoexcited states.^73^ Due to the different work function of the participating semiconductors, a strong electric field is induced.^74^

**Figure 10:**
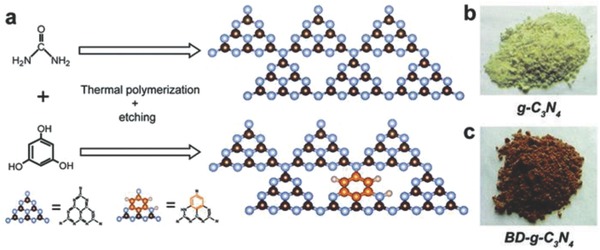
a) Schematic view of the construction, and b,c) optical photographs of g‐C_3_N_4_ and BD‐C_3_N_4_. Adapted with permission from.^75^ Copyright 2017, the Royal Society of Chemistry.

A new approach beyond doping and heterojunctions has been conceptualized to deal with charge transfer kinetics in CN. This was realized by aromatic ring substitution where a benzene ring was substituted in triazine‐based CN (see **Figure** **10**).^75^ Two main advantages arise from this. First, it improves light absorption by bringing the doped band into the forbidden gap. Second, the introduction of all‐carbon aromatic ring (i.e., benzene ring) acts as an electron buffer, which greatly expedites transfer of the excited π‐electron delocalization in the conjugated system. This therefore inhibits the recombination of electrons with holes. As a result, a controlled amount of benzene ring substituted CN evolved four times greater hydrogen production than that of pristine CN.

Recently, Rahman et al. reported polymeric hybrid heterojunction that in situ coupled polymeric carbon nitride onto amorphous carbon nitride.^76^ This hybrid photocatalyst showed record high quantum yield of 5% at 420 nm of visible light irradiation in the absence of noble metal co‐catalyst. While carbon nitride is highly dependent on the precious metal catalyst (e.g., Pt) for enhancing the quantum yield, this result is indeed a breakthrough.

## 5 Concerns with Using SED for Hydrogen Evolution and Reproducibility of AQE

Carbon nitrides have well‐defined CB and VB positions for water splitting that enable them to be used for hydrogen evolution either through a water‐splitting half‐reaction or overall water splitting. A complete water‐splitting reaction involves two half‐reactions. One for proton reduction to hydrogen (known as a reduction reaction, requiring two electrons) and the other the water oxidation reaction to produce oxygen (known simply as oxidation, requiring four electrons). Carbon nitride is thermodynamically unstable for water oxidation. Therefore the oxidation reaction is more physiochemically challenging and sets a thermodynamic limit. It is because the carbon in carbon nitrides is less electronegative than the oxygen that fundamentally limits its application.^13^, ^15^, ^31^, ^77^

There are three process terms, namely, photocatalytic, photosynthetic, and artificial photosynthesis, which are used synonymously in the literature for hydrogen evolution via water splitting. However, there are fundamental thermodynamic differences in these processes. Photocatalytic processes are thermodynamically downhill that promote reactions with Δ*G* < 0 without storage of photochemical energy, while photosynthetic (including artificial photosynthesis) devices use the energy of light to drive reactions with Δ*G* > 0. Unlike a photocatalytic system, a photosynthetic system must suppress the reverse photosynthetic reaction.^78^

There are two types of strategies for preventing reverse reactions in photosynthetic systems, Type 1 and Type 2. In Type 1, the back reaction is prevented by spatially separated cathodic and anodic half‐reactions, whereas in Type 2 it is accomplished by charge‐transfer selectivity. Therefore, under optimized conditions (large absorption coefficients, long carrier lifetimes, fast interfacial charge‐transfer kinetics), photocatalytic processes are limited by surface area, while photosynthetic processes are limited by charge mobility and mass transport (Type 1) and by electrochemical selectivity (Type 2).^78^

When hydrogen production is the sole purpose, researchers use SED to suppress one half‐reaction and promote only the hydrogen evolution reaction. However, photocatalytic water splitting (thermodynamically forbidden) and photocatalytic hydrogen evolution from solutions of sacrificial electron donors (thermodynamically favored) are different reactions. Hydrogen production in the presence of SED, e.g., TEOA, is a thermodynamically downhill process. The presence of this electron donor speeds up the hole transfer from carbon nitride, and consequently reduces the positive charging of the material. Moreover, addition of TEOA increases the alkalization (i.e., pH) of the solution. The increase in pH shifts the quasi‐Fermi level in GCN to a more reducing value and therefore increases hydrogen evolution.^79^ The SED can scavenge the holes and therefore the charge carrier recombination can be greatly reduced. As oxygen is not produced, the added advantage is the blocking of the back reaction to produce water. This ultimately increases the hydrogen yield and obviates a subsequent gas‐separation stage. Most research is therefore directed to produce hydrogen via the water‐splitting half‐reaction while little success has been achieved with overall water splitting.

It is to be noted that if there is no photocatalyst, SED alone cannot produce hydrogen from water. Moreover, there is a chance of a subsequent decrease in yield of hydrogen formation by competing reduction reactions with the products formed on oxidation of the sacrificial reagents.^80^ The concern is that the high product quantum yield of hydrogen reportedly produced in the presence of SED does not imply hydrogen production from overall water splitting. There is, in consequence, the need for research into overall water splitting in the absence of SED.

There is also a growing concern about the reproducibility of the quantum yield of hydrogen (i.e., AQE) for a comparison of photocatalytic activities. As the rate of photoreaction is proportional to the number of photons absorbed at a given wavelength per unit of time and volume,^81^ therefore comparison of the results is meaningful only if the fraction of light absorbed is the same in each experiment. Complicating this issue, the amount of scattered and reflected light might change significantly from one experiment to another.^82^

The product quantum yield in photocatalytic water splitting is simultaneously affected by the efficiency of the i) formation of reactive electron–hole pairs, ii) interfacial electron transfer, and iii) product formation from the primary redox products.^3^ If there is any change in these production rates induced by a given photocatalyst, it is difficult to identify which of these efficiencies is responsible for the observed changes.

The quantum yield of hydrogen is strongly influenced by the wavelength and the intensity of the irradiating light. When the irradiation wavelength approaches the absorption edge, this lowers the absorption coefficients and increases the migration distances for photoexcited carriers. This problem can be overcome using a monochromatic light source. Similarly, charge recombination is a second‐order reaction with increasing light intensity. These cumulatively decrease the quantum yield of hydrogen.^83^ Therefore, reporting of AQE values together with the intensity and the distribution of the incident light is highly recommended.

Care should also be taken in reporting the hydrogen production rate. The production rate should not be normalized with the mass of the photocatalyst. This is because photocatalytic rates are not proportional to the mass of the photocatalysts due to saturation of light absorption at some point. Therefore using mol h^−1^ g^−1^ is misleading.^84^ Accordingly, AQE should be measured only when the amount of photocatalyst is optimum for saturated light absorption.^83^

## 6 Conclusions and Future Perspective

Carbon nitride is a versatile energy material. Despite the limitation of defect‐free polymeric carbon nitride syntheses, it has unique physicochemical properties and has therefore been studied extensively since 2009 for photocatalytic hydrogen production via water splitting. Attempts have been made to enhance its photocatalytic performance by modulation of intrinsic and extrinsic activities. Although most research has been made to improve its extrinsic activities, the resulting apparent quantum efficiency remains moderate at less than 10%. This has motivated research to enhance intrinsic activities, by molecular engineering and chemical modification, to improve catalytic activity. This has been accomplished based on a molecular level understanding attained via computational quantum chemistry and experimental tailoring of chemical composition and physical structure through customized nanostructuring. With tailored molecular tuning, the AQE of carbon nitride is reported as great as 60% in the presence of SED. It is concluded therefore that molecular tuning of intrinsic activities has begun a new paradigm in research to overcome limited success by extrinsic activities.

Any increase in quantum efficiency of carbon nitride requires minimum photon loss, meaningful delocalization of the exciton to avoid extrinsic recombination, and leveraging of catalytic active sites and electron utilization for proton reduction. The greatest challenge is efficient charge separation. There is a direct link between charge carrier dynamics and photocatalytic efficiency. For example, a photocatalyst with a narrow absorption band, but efficient charge transport, will result in greater photocatalytic efficiency than a photocatalyst with an extended absorption edge, but poor charge transport. Therefore, an atomistic investigation of the dynamics (recombination, separation, and migration) of photogenerated charge carriers will be vital for understanding photophysical parameters that control photoactivity.

**Figure 11:**
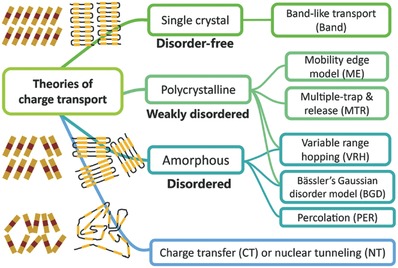
Charge transport theories and selection of charge transport model for organic semiconductors. Reproduced with permission. Copyright 2017, the Royal Society of Chemistry.

However, little is known about the fundamental charge transport dynamics that are needed to explain photophysical processes in carbon nitrides. As is depicted in **Figure** **11**, there are several models to describe microscopic understanding of charge transport. Selection of a specific model depends on the crystal structure and atomic arrangement of a given carbon nitride. The explanation of charge transport in carbon nitrides is presently vague and superficial. The adoption of a well‐defined charge transport model is needed. A generalized model is necessary to describe, predict, and compare charge transport in carbon nitrides.

Photogenerated charge carriers have finite mobility and lifetime, depending on the material, the carrier type, and the light intensity. To drive water redox reactions, these carriers need to reach the material's interfaces at the electrolyte and at the co‐catalysts. Charge carriers can move either by drift due to the presence of a bias voltage/external electric field (for the case of a photoelectrochemical cell) or by diffusion only (for the case of a photocatalyst) in the absence of an external field.^85^ However, charge carrier mobility and diffusion lengths are not known for electrons and holes in carbon nitrides, even though these parameters are critical for solar fuel applications of this material.

The lifetime of carriers generated in carbon nitride by absorption of photons is less than a nanosecond. The carrier lifetime needs to be increased to the millisecond timescale to compete with the faster rate of recombination. This substantial improvement in lifetime requires increased spatial separation of charge carriers. One possible way to achieve this is to use redox co‐factors, for example, donor–acceptor cascaded systems.^86^ The redox co‐factors undertake a vectorial series of electron transfer events—with each forward step proceeding over a short distance that results in spatial separation of short‐lived molecular excited states into long‐lived charge separated states. Due to this spatial separation, electrons need to tunnel a long distance to recombine with the holes. This rate of electron tunneling decays exponentially with distance and, therefore, nullifies the chance of electron–hole recombination. This could occur with a spatial separation of only a couple of nanometers.^87^ Each forward electron transfer will then be several orders of magnitude faster than the competing recombination pathways to ground.

The dual synergistic action of increasing optical absorption and creating hot electrons could be obtained through a heterojunction composed of nanometer size metallic clusters (e.g., Au and Ag) on carbon nitrides.^88^ The inclusion of metal clusters to act as optical antennas with large absorption cross‐sections would allow concentration of light at the nanometer scale.^89^ This might also lead to single‐particle excitation through momentum scattering to create a homogeneous distribution of hot electrons either in the metal, at the interface, or in the host semiconductor. These hot electrons are useful for catalyzing chemical reactions.^90^ However, because of the ultrafast decay, the generation, transport, and relaxation of hot electrons is poorly understood.

Moreover, researchers may try to implement localized photon absorption (Anderson's effect), multiple excitation generation, singlet fission, upconversion, superlattices, etc., which have been proven effective for photovoltaic solar energy conversion.^91^ This will therefore require an adroit manipulation of the carbon nitride electronic structure via quantum confinement and new charge carrier dynamics.

The practical demonstration of solar energy conversion via photovoltaic cells and photoelectrochemical cells began almost at the same time.^92^ While solar cell technologies are already commercialized, researchers, despite an intervening four decades, are struggling to find a suitable material for stable and efficient photocatalytic generation of solar fuel. For economically viable hydrogen production, the photocatalyst must be developed from inexpensive semiconductor(s) that can stably operate continuously on a timescale of years. It is worth mentioning that the current best performing photocatalyst materials are not stable, even for a month, and consist of toxic elements. Carbon nitride has only moderate conversion efficiency; however, a major benefit is that it is an earth‐abundant semiconductor that has long‐term stability.

Most photovoltaic technology is not based on high‐performing devices—as these are far too expensive. Similarly, although carbon nitride has only a moderate photocatalytic performance at present, with molecular tuning it could possibly reach the Shockley–Queisser limit, and we propose therefore it could be used as a model photocatalyst in industrial‐scale hydrogen production. However, this may not be straightforward to implement.

## Conflict of Interest

The authors declare no conflict of interest.
